# Assessment of Intakes and Patterns of Cooked Oatmeal Consumption in the U.S. Using Data from the National Health and Nutrition Examination Surveys

**DOI:** 10.3390/nu8080503

**Published:** 2016-08-17

**Authors:** Kathy Musa-Veloso, Shafagh Fallah, Marianne O’Shea, YiFang Chu

**Affiliations:** 1 Intertek Scientific & Regulatory Consultancy, Mississauga, ON L5N 2X7, Canada; kathy.musa-veloso@intertek.com (K.M.-V.); shafagh.fallah@intertek.com (S.F.); 2 Quaker Oats Center of Excellence, PepsiCo R&D Nutrition Sciences, Barrington, IL 60010, USA; marianne.oshea@pepsico.com

**Keywords:** oatmeal, intake, ingestion, cereal, NHANES, whole grains

## Abstract

The objective of the present study was to characterize the consumption of cooked oatmeal in the United States (U.S.) and to determine whether oatmeal consumption is associated with body mass index (BMI). To estimate current intakes of cooked oatmeal in the various age and gender population groups, we used dietary intake data from Day 1 of the U.S. 2009–2010 and 2011–2012 National Health and Nutrition Examination Surveys (NHANES). We also used dietary intake data from Day 1 of the U.S. 2003–2012 NHANES to assess associations between intakes of cooked oatmeal (in g/kg body weight) and NHANES cycle (2003–2004, 2005–2006, 2007–2008, 2009–2010, 2011–2012), age category (3–11 years, 12–18 years, 19–44 years, 45 years+), gender, and BMI classification (underweight, normal weight, overweight, or obese), using a multiple linear regression model. A consumer of oatmeal was defined as any individual who reported the consumption of any amount of oatmeal on Day 1 of the survey. Approximately 6% of the total population consumed oatmeal, with an average intake of 238 g/day of cooked oatmeal among consumers. The greatest prevalence of oatmeal consumption was in infants (14.3%) and older female adults (11.1%). Amongst oatmeal consumers, underweight, normal weight, and overweight individuals consumed significantly more oatmeal than obese individuals. Oatmeal was consumed almost exclusively at breakfast and, among consumers, contributed an average of 54.3% of the energy consumed at breakfast across all age groups examined. The association between oatmeal consumption and BMI is interesting and requires confirmation in future clinical studies.

## 1. Introduction

Oatmeal is a common whole-grain cereal that is rich in β-glucan, a soluble fiber that has multiple functional and bioactive properties. The consumption of oat β-glucan has been associated with reductions in low-density lipoprotein (LDL)-cholesterol [1,2] and postprandial glucose and insulin levels [3], as well as improvements in subjective measures of appetite [4]. In a study of children aged 2–18 years who had participated in the 2001–2010 United States (U.S.) National Health and Nutrition Examination Surveys (NHANES), those who were oatmeal consumers had better overall diet quality and reduced risks of central adiposity and obesity compared with children who were non-consumers of oatmeal [5]. Likewise, in a study of adults aged 19 years or older who had participated in the 2001–2010 U.S. NHANES, those who were oatmeal consumers had better overall diet quality and lower body weights, waist circumferences, and body mass indices (BMIs) compared with adults who were non-consumers of oatmeal [6]. Therefore, promoting oatmeal consumption may help improve health, and understanding patterns of oatmeal consumption in the U.S. according to age, gender, and BMI can help target those individuals who may benefit from the incorporation of oatmeal into the diet.

In addition to increasing the dietary fiber content of the diet and improving satiety, the consumption of oatmeal may result in a reduction in the energy density of the overall diet which, in turn, may be useful in the management of body weight. The Institute of Medicine (IOM) has established adequate intake (AI) levels for dietary fiber for the various age and gender groups. Accordingly, the recommended intakes for dietary fiber are 19 to 25 g/day for children ages 1–8 years, 26–38 g/day for children and adolescents aged 9–18 years, and 21–38 g/day for adults aged 19 years or older [7]. Despite these recommendations, the consumption of dietary fiber is notably low in the U.S. Based on data from the 2001–2010 NHANES, McGill et al. [8] reported that the mean intakes of dietary fiber were 13.2, 16.1, and 16.1 g/day in children and adolescents aged 4–18 years, adults aged 19–50 years, and adults 51 years of age and older, respectively. These intakes are approximately half of the intakes that are recommended by the IOM and that are endorsed in the 2015 Dietary Guidelines for Americans [9]. 

In an analysis of the 2009–2010 NHANES data, it was demonstrated that whole-grain cereal was the greatest contributor to dietary fiber intake among individuals with the highest intake of whole grains and that individuals who consumed at least three ounces of whole grains per day were approximately 60–75 times more likely to be in the top tertile of fiber consumption [10]. Therefore, encouraging the consumption of whole-grain cereals may help individuals reach an adequate daily intake of dietary fiber [7]. 

In the present study, intakes of cooked oatmeal using Day 1 of the 2009–2010 and 2011–2012 NHANES were analyzed to investigate trends in the consumption of cooked oatmeal according to age, gender, and BMI. In addition, a longitudinal analysis was performed by comparing trends in oatmeal consumption, using data from the previous five NHANES cycles (2003–2004, 2005–2006, 2007–2008, 2009–2010, and 2011–2012). Cooked oatmeal is expected to induce a greater viscosity in the upper gastrointestinal system than other oat-based products; thus, the intake of cooked oatmeal, in particular, was of interest, and not the intake of oats used in other applications, such as in muffins, breakfast bars, granola, etc.

## 2. Materials and Methods

### 2.1. Survey Description

Cooked oatmeal consumption patterns were analyzed using the 2009–2010 and 2011–2012 NHANES, which are continuous, cross-sectional, population-based surveys that include data on the nutritional and health status of the U.S. population using a representative, multistage, probability sampling design. Detailed descriptions of survey methods and sampling design have been previously described [11]. In brief, NHANES is conducted by the National Center for Health Statistics (NCHS), and participants complete questionnaires that assess dietary behaviors, health history, socioeconomic status, and other demographic information. The NCHS Research Ethics Review Board reviewed and approved all study protocols for NHANES. For the 2009–2010 NHANES, 13,272 individuals were screened, of whom 10,537 (79.4%) were interviewed and 10,253 (77.3%) were examined. For the 2011–2012 NHANES, 13,431 individuals were selected, of whom 9756 (72.6%) were interviewed and 9338 (69.5%) were examined.

### 2.2. Dietary Assessment

Dietary intake data were collected via 24-h dietary recalls administered on two non-consecutive days (termed Day 1 and Day 2). Day 1 data were collected in person, and Day 2 data were collected by telephone 3–10 days later on a different day of the week. Dietary recall required participants to describe details of all foods and beverages consumed over the previous 24 h, including the time of consumption, the name of the eating occasion (i.e., breakfast, lunch, dinner, snack, or infant feeding), detailed food descriptions, and the amounts of foods consumed. For survey participants younger than six years of age, interviews were conducted with a proxy (who was generally the person most knowledgeable about the survey participant’s intake). For children aged 6–8 years, interviews were conducted with a proxy and with the child present to assist in reporting intake information. For children 9–11 years of age, interviews were conducted with the child and the assistance of an adult familiar with the child’s intake. Participants 12 years of age or older reported their own dietary intakes. Dietary interviewers conducted interviews in English and Spanish, with translators used to conduct interviews in languages other than English and Spanish.

NHANES food codes representative of the consumption of cooked oatmeal were included in the assessment of dietary intakes (Table 1). As can be seen from Table 1, each food code was representative of the intake of cooked (reconstituted) oatmeal, except for code 57804000 (oatmeal cereal, baby food, dry, instant), code 57806100 (oatmeal cereal with bananas, baby food, dry, instant), and code 57806200 (oatmeal cereal with fruit, baby food, dry, instant, toddler). For these three food codes, the amount of cooked (reconstituted) oatmeal that would have been consumed, based on the amount of dry oatmeal that was reported, was estimated by adding 110 mL of water to every 15 g of dry oatmeal (i.e., the conversion factor applied to the dry oatmeal was 8.3). All of the oatmeal intakes presented herein are of cooked (reconstituted) oatmeal; intakes of oats used in other applications, such as in muffins, breakfast bars, granola, etc., were not considered. The oatmeal intakes presented herein are based only on intake data reported on Day 1 of the survey. Thus, a consumer of cooked oatmeal was defined as any individual who reported the consumption of any amount of cooked oatmeal on Day 1 of the survey. In an individual identified as a consumer of cooked oatmeal, the total intake of cooked oatmeal was defined as the total amount of cooked oatmeal that was consumed on Day 1 of the survey, irrespective of the eating occasion. Intakes of cooked oatmeal at each eating occasion (i.e., breakfast, lunch, dinner, snack, infant feeding) also were examined and are presented.

The intakes of oatmeal, including the prevalence of consumption and the mean, 90th percentile, and range of consumption (in grams, kcal, and as a proportion of energy consumed), among respondents identified as consumers of oatmeal are presented for each population group (i.e., all youths (0–18 years), infants (0–2 years), children (3–11 years), male teens (12–18 years), female teens (12–18 years), all adults (19 years+), male adults (19–44 years), female adults (19–44 years), male adults (45 years+), female adults (45 years+), and the total population (all ages)). Appropriate sample weights were applied in all of the analyses. 

The distribution of oatmeal consumers by age (ungrouped) was examined. In addition, the top five foods that were most frequently consumed at breakfast when oatmeal also was consumed at breakfast were identified. As a comparison, the top five foods that were most frequently consumed at breakfast in individuals who were not consumers of cooked oatmeal also were examined.

### 2.3. Prevalence of Oatmeal Consumption—A Longitudinal Assessment

The prevalence of oatmeal consumption for each population group (i.e., infants (0–2 years), children (3–11 years), male teens (12–18 years), female teens (12–18 years), male adults (19–44 years), female adults (19–44 years), male adults (45 years+), female adults (45 years+), and the total population (all ages)) was assessed for five NHANES cycles (2003–2004, 2005–2006, 2007–2008, 2009–2010, and 2011–2012). Trends were examined visually, and NHANES cycle was included as an independent variable in the multiple linear regression model (described in Section 2.4).

### 2.4. Assessment of the Associations of Independent Variables and Oatmeal Intake

A multiple linear regression model was used to examine the association between oatmeal consumption (total from all eating occasions combined, relative to body weight (in g/kg body weight)) and four main variables, namely (i) NHANES cycle (2003–2004, 2005–2006, 2007–2008, 2009–2010, 2011–2012); (ii) age category (3–11 years, 12–18 years, 19–44 years, 45 years+); (iii) gender; and (iv) BMI classification (underweight, normal weight, overweight, or obese). The BMI of each individual three years of age or older who was identified as an oatmeal consumer was classified using the guidelines of the U.S. Centers for Disease Control and Prevention (CDC) [12]. Adults (19 years+) were classified as either underweight (BMI < 18.5 kg/m^2^), normal weight (BMI 18.5–24.9 kg/m^2^), overweight (BMI 25–29.9 kg/m^2^), or obese (BMI ≥ 30 kg/m^2^). For children 3–18 years, BMI classifications were based on the CDC classifications for age and gender percentiles [13]. It should be noted that, according to the CDC classifications, the categorization as underweight, normal weight, overweight or obese starts from age three years; hence, the multiple linear regression assessment was restricted to individuals aged three years and older. 

### 2.5. Statistical Analysis

Statistical analyses were conducted using SAS software (version 9.4, SAS Institute Inc., Cary, NC, USA) and SUDAAN software (version 11.0.1, Research Triangle Institute, Research Triangle Park, Durham, NC, USA). For the multiple linear regression, proc regress in SUDAAN was used. Statistical significance was defined as an alpha level of *p* < 0.05.

## 3. Results

### 3.1. Prevalence of Cooked Oatmeal Consumption

The total intakes of cooked oatmeal (in g/day) from all eating occasions (i.e., breakfast, lunch, dinner, snack, and infant feeding) are summarized in Table 2. Of youths aged 0–18 years, the greatest prevalence of oatmeal consumption occurred in infants aged 0–2 years, of whom 14.3% were reported to have consumed oatmeal on Day 1 of the 2-day NHANES (2009–2012). Of adults aged 19 years or older, the greatest prevalence of oatmeal consumption occurred in female adults aged 45 years or older, of whom 11.1% reported the consumption of oatmeal on Day 1 of the two-day NHANES (2009–2012). Among all youths (0–18 years), all adults (19 years of age or older), and the total population, the prevalence of oatmeal consumption on Day 1 of the two-day NHANES (2009–2012) was 4.6%, 6.5%, and 6.0%, respectively. The corresponding mean intakes of cooked oatmeal from all eating occasions, combined, were 182, 251, and 238 g/day, respectively (Table 2) (for reference, one cup of cooked oatmeal is approximately 234 g).

**Figure 1:**
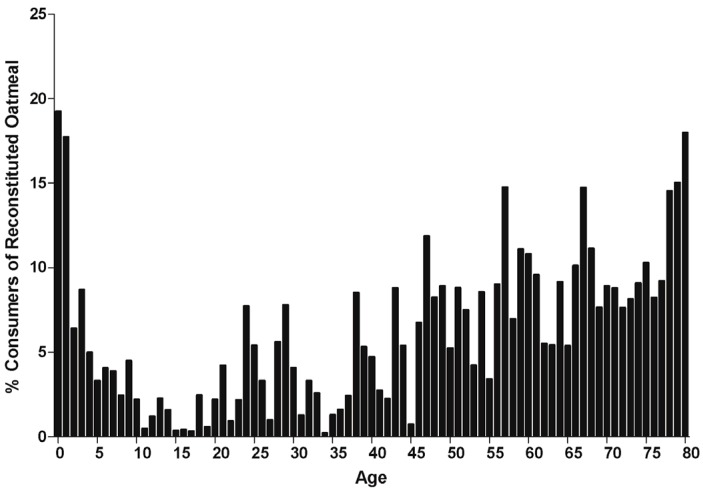
Distribution of consumers of cooked oatmeal according to the proportion of consumers within each age group. Percentage (%) of total number of respondents within an age year who were identified as consumers of cooked oatmeal within the 2009–2012 NHANES. A respondent was considered a consumer of cooked oatmeal if they consumed any of the foods represented by the food codes listed in Table 1 on Day 1 of the survey. It should be noted that individuals aged 80 years or older are top coded at 80 years of age.

The distribution of individuals reporting the consumption of oatmeal according to age approximated a U- or J-shaped pattern, with a high proportion of oatmeal consumers in the infant (0–2 years) and older adult (45 years or older) age groups and lower proportions of oatmeal consumers in the children, teenagers, and younger adult age groups, respectively (Figure 1). It should be noted that individuals aged 80 years or older are top-coded at 80 years of age, and so their intakes are captured as intakes at 80 years of age.

### 3.2. Oatmeal Consumption According to Eating Occasion

The number of consumers who reported the consumption of cooked oatmeal at the various eating occasions (i.e., breakfast, lunch, dinner, snack, or infant feeding), as well as the associated intakes (in g/day) of cooked oatmeal, are summarized in Table 3. For all age and gender groups, oatmeal was consumed predominantly at breakfast. Of youths aged 0–18 years, the greatest prevalence of oatmeal consumption at breakfast occurred in infants aged 0–2 years, of whom 9.6% were reported to have consumed oatmeal at breakfast on Day 1 of the two-day NHANES (2009–2012). Of adults aged 19 years or older, the greatest prevalence of oatmeal consumption at breakfast occurred in female adults aged 45 years or older, of whom 10.3% reported the consumption of cooked oatmeal at breakfast on Day 1 of the two-day NHANES (2009–2012). Among all youths (0–18 years), all adults (19 years of age or older), and the total population, the prevalence of oatmeal consumption at breakfast on Day 1 of the two-day NHANES (2009–2012) was 3.7%, 5.8%, and 5.3%, respectively. The corresponding mean intakes of cooked oatmeal at breakfast were 185, 243, and 232 g/day, respectively (for reference, one cup of cooked oatmeal is approximately 234 g). For the lunch, dinner, and snack eating occasions, the prevalence of oatmeal consumption ranged from 0% to 0.5% of individuals surveyed for all age and gender groups, except for infants, for whom 0.8%, 1.3%, and 1.2% of those surveyed were reported to have consumed oatmeal at lunch, dinner, or as a snack, respectively. Also, 3.0% of the infants surveyed were reported to have consumed oatmeal at an “infant feeding” occasion that was not characterized as breakfast, lunch, dinner, or snack.

### 3.3. Intake of Energy from Cooked Oatmeal at Breakfast

Given that oatmeal was found to be consumed predominantly at breakfast, the intake of energy from oatmeal, as well as the contribution of oatmeal to the total intake of energy consumed at breakfast were assessed (Table 4). Male teens aged 12–18 years and male adults aged 19–44 years were found to have the greatest mean intakes of energy at breakfast from oatmeal, at 266.1 and 267.1 kcal, respectively. Among all youths (0–18 years), all adults (19 years of age or older), and the total population, the intake of energy from the consumption of oatmeal at breakfast on Day 1 of the two-day NHANES (2009–2012) was 162.9, 200.3, and 193.7 kcal, respectively. The contribution of oatmeal to the intake of energy at breakfast ranged from 44.2% to 74.0% at the mean; however, for some individuals in all age groups (except female teens), oatmeal contributed to 100% of the total intake of energy at breakfast on Day 1 of the two-day NHANES.

### 3.4. Food Choices at Breakfast of Oatmeal Consumers and Non-Consumers

In Table 5, the foods that were co-consumed with oatmeal in individuals for whom oatmeal contributed only to a portion of the energy intake consumed at breakfast are presented. In individuals 18 years and younger, milk/yoghurt was generally the food that was the most frequently consumed with oatmeal at breakfast, followed by fruit juices and fruits. In adults 19 years and older, sugars and sweets were the most commonly consumed foods with oatmeal at breakfast, followed by coffee or tea, fruits, and milk/yoghurt.

In Table 6, the foods that were most frequently consumed at breakfast in individuals who did not consume cooked oatmeal at breakfast are presented. In individuals 18 years and younger, milk/yoghurt was the food that was consumed most frequently, followed by non-oatmeal cereals, and fruit juices. In adults 19 years and older, the most commonly consumed foods were coffee and tea, followed by milk/yoghurt, sugars and sweets, yeast breads, rolls, quick breads, and non-oatmeal cereals.

### 3.5. Longitudinal Assessment of Oatmeal Intake

**Figure 2:**
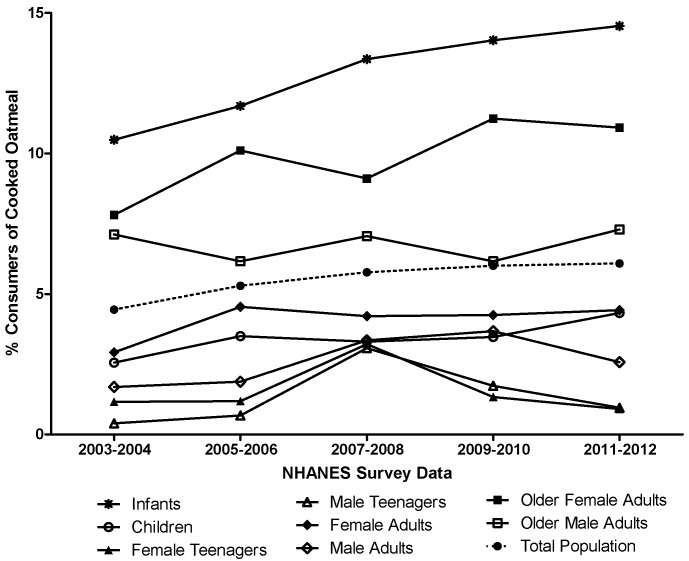
Percent of each population group identified as consumers of cooked oatmeal. Percentage of respondents identified as consumers of cooked oatmeal within the 2003–2004, 2005–2006, 2007–2008, 2009–2010, and 2011–2012 cycles of the NHANES. A respondent was considered a consumer of cooked oatmeal if they consumed any of the foods represented by the food codes listed in Table 1 on Day 1 of the survey.

Among the total population, the percentage of oatmeal consumers remained fairly stable from 2003 to 2012 (Figure 2); in contrast, the percentage of oatmeal consumers increased over time in infants and older female adults and was always greater in these population groups than in the total population (Figure 2). The association between oatmeal consumption (total intake on Day 1, expressed relative to body weight (i.e., g/kg body weight)) in oatmeal consumers and several different variables (namely, age category, gender, NHANES cycle, and BMI category) was assessed using a linear regression model (Table 7). It should be noted that infants were removed from this assessment, given that reference data for the classification of individuals as underweight, normal weight, overweight, or obese are available only for individuals three years of age or older. With regards to gender, males consumed more oatmeal than did females, with the difference nearly approaching statistical significance (*p* = 0.06). With regards to BMI status, underweight, normal weight, and overweight individuals consumed significantly more oatmeal than did obese individuals. With regards to age, children, teenagers, and young adults consumed significantly more oatmeal than did older adults. The NHANES cycle was not associated with oatmeal intakes. With the inclusion of all of these variables in the model, 39% of the variability in oatmeal consumption was explained.

## 4. Discussion

Oatmeal contains the soluble fiber β-glucan, which has physiological and bioactive properties that may help in improving blood lipid levels, postprandial insulin, and glucose responses, and subjective measures of satiety. Therefore, understanding the current trends of oatmeal consumption in the U.S. may help in the development of targeted interventions to increase consumption among particular age, gender, and BMI groups. We analyzed data from the 2009–2010 and 2011–2012 NHANES to better understand the consumption of cooked oatmeal in the U.S. Limitations to our study include the cross-sectional design of NHANES, which limits our ability to establish a causal relationship between independent variables and oatmeal intake, and the small sample sizes in some of our groups. Additionally, we assessed oatmeal intakes based on one 24-h recall, which may not reflect the typical dietary patterns of the participants. Furthermore, food intake was self-reported by the participants or their caretakers, who may have under- or over-reported food consumption. Nevertheless, the NHANES is based on a nationally representative random sample of the U.S. population and includes numerous variables, which are key strengths of the survey.

Overall, we found a low prevalence of oatmeal consumption (6.0% of the total population surveyed consumed cooked oatmeal on Day 1 of the survey, Table 2), especially in children, teens, and young adults (19–44 years). Moreover, only 5.3% of the individuals surveyed included cooked oatmeal in their breakfast meal. In our study, oatmeal accounted for approximately 54.3% of the total energy intake at breakfast, indicating that most respondents consumed other food items along with oatmeal for their breakfast meal. The foods that were most commonly co-consumed with oatmeal were milk/yoghurt, fruit juice, and fruit in children and sugars/sweets, coffee/tea, fruits, and milk/yoghurt in adults. In individuals who were not oatmeal consumers, the most commonly consumed foods at breakfast were milk/yoghurt, cereals, fruit juices, and yeast breads/rolls/quick breads in children and coffee/tea, milk/yoghurt, sugars/sweets, and yeast breads/rolls/quick breads in adults. Interestingly, in both adult consumers and non-consumers of oatmeal, the most commonly consumed foods included coffee/tea, milk/yoghurt, and sugars/sweets. Thus, it is likely that sugars/sweets and milk were used in the preparation of coffee/tea in both adult groups. Non-oatmeal cereals and yeast breads/rolls/quick breads were amongst the top five most commonly consumed foods at breakfast in youth and adult non-consumers of oatmeal. Interestingly, fruits were one of the top five foods consumed by adult and youth oatmeal consumers; however, in non-oatmeal consumers, fruits were not one of the top five most commonly consumed foods at breakfast, neither in children nor in adults (fruits ranked as the eighth and ninth most commonly consumed foods in children and adults, respectively). This observation seems consistent with the findings that in children aged 2–18 and adults aged 19 years or older who participated in NHANES (2001–2010), cooked oatmeal consumption was associated with better diet quality [5,6].

In a multiple linear regression analysis, oatmeal consumption (intakes on Day 1 of the survey, standardized according to body weight) amongst oatmeal consumers was found to be significantly greater in underweight, normal weight, and overweight individuals compared to obese individuals. Based on data from 2001–2010 NHANES, it was reported that children aged 2–18 years and adults aged 19 years or older who consumed oatmeal had more favorable body weights/compositions than individuals who did not consume oatmeal [5,6]. In another analysis of NHANES data (1999–2004), it was noted that breakfast consumption was associated with a lower BMI in adult women but not adult men [14,15], and that consumption of ready-to-eat or cooked cereal was associated with a lower BMI and waist circumferences in both genders [16]. 

Previous studies indicate that increasing the consumption of cereals, including oatmeal, is associated with favorable effects on BMI. In a systematic review of 14 studies, cereal consumption at breakfast was associated with a small but significant decrease in BMI in children and adolescents [17]. In another systematic review investigating breakfast cereal consumption in adults, there was grade B evidence that regular breakfast cereal consumption is associated with a lower BMI and reduced risk for overweight and obesity [18].

Our results indicate that the prevalence of oatmeal consumption at an eating occasion other than breakfast is very low. Other than in infants, the prevalence of consumption of oatmeal at lunch, dinner, or as a snack was 0.5% or less. In several studies, it has been suggested that the consumption of cereals at a meal other than breakfast may be useful for weight management. Mattes [19] demonstrated that when cereal was consumed at breakfast and at either lunch or dinner, there was a greater loss in body weight than when individuals were not given any dietary recommendations to replace meals with cereals. Similarly, Waller et al. [20] reported improved weight loss in individuals who consumed cereal with low-fat milk as a late evening snack compared to controls who were not given any dietary recommendations on what to consume for their late-night snack. 

Consumption of oatmeal may help with feeling less hungry, as oatmeal consumption reduced subjective measures of appetite more than a ready-to-eat breakfast cereal in a randomized cross-over trial [21] and consumption of oat β-glucan increased levels of the hunger-suppressing hormone cholecystokinin and decreased subsequent meal intake [3]. These effects are likely to be due, in part, to the high viscosity of β-glucan, which leads to decreased hunger and increased fullness, perhaps by prolonging the transit time and absorption rate of nutrients [22]. Further research should investigate whether there is any causal relationship between oatmeal consumption and BMI or other indicators of health status among children and adults, to validate the favorable associations noted in our study, other cross-sectional studies, and in clinical studies of other cereals. In addition, it is important to determine whether the viscosity the cereal induces in the upper gastrointestinal system is an important determinant of its potential efficacy. In our analysis, we looked only at the intakes of cooked oatmeal, and cooked oatmeal is expected to induce a greater viscosity in the upper gastrointestinal system than other oat-based products, such as cookies, muffins, granola, and muesli.

## 5. Conclusions

Based on dietary intake data from Day 1 of the 2011–2012 U.S. NHANES, approximately 6% of the total population consumed oatmeal, with an average intake of 238 g/day of cooked oatmeal among consumers. Oatmeal was consumed almost exclusively at breakfast and, among consumers, contributed an average of 54.3% of the energy consumed at breakfast across all age groups examined. Based on dietary intake data from Day 1 of the 2003–2012 NHANES, BMI status was significantly associated with oatmeal consumption among oatmeal consumers, such that underweight, normal weight, and overweight individuals consumed significantly more oatmeal than obese individuals. The association between oatmeal consumption and BMI is interesting, and is consistent with clinical studies in which the consumption of oatmeal was found to induce improvements in subjective measures of satiety. Future clinical studies are needed to determine the effects of regular oatmeal consumption on body weight.

## Figures and Tables

**Table 1:** Food codes representative of cooked oatmeal within the 2003–2012 NHANES databases.

Food Code	Description
56202960	Oatmeal, cooked, NS as to regular, quick or instant; NS as to fat added in cooking
56202970	Oatmeal, cooked, quick (1 or 3 min), NS as to fat added in cooking
56202980	Oatmeal, cooked, regular, NS as to fat added in cooking
56203000	Oatmeal, cooked, NS as to regular, quick or instant, fat not added in cooking
56203010	Oatmeal, cooked, regular, fat not added in cooking
56203020	Oatmeal, cooked, quick (1 or 3 min), fat not added in cooking
56203030	Oatmeal, cooked, instant, fat not added in cooking
56203040	Oatmeal, cooked, NS as to regular, quick, or instant, fat added in cooking
56203050	Oatmeal, cooked, regular, fat added in cooking
56203060	Oatmeal, cooked, quick (1 or 3 min), fat added in cooking
56203070	Oatmeal, cooked, instant, fat added in cooking
56203080	Oatmeal, cooked, instant, NS as to fat added in cooking
56203110	Oatmeal with maple flavor, cooked
56203200	Oatmeal with fruit, cooked
56203210	Oatmeal, NS as to regular, quick, or instant, made with milk, fat not added in cooking
56203211 ^a^	Oatmeal, cooked, regular, made with milk, fat not added in cooking
56203212 ^a^	Oatmeal, cooked, quick (1 or 3 min), made with milk, fat not added in cooking
56203213 ^a^	Oatmeal, cooked, instant, made with milk, fat not added in cooking
56203220	Oatmeal, NS as to regular, quick, or instant, made with milk, fat added in cooking
56203221 ^a^	Oatmeal, cooked, regular, made with milk, fat added in cooking
56203222 ^a^	Oatmeal, cooked, quick (1 or 3 min), made with milk, fat added in cooking
56203223 ^a^	Oatmeal, cooked, instant, made with milk, fat added in cooking
56203230	Oatmeal, NS as to regular, quick, or instant, made with milk, NS as to fat added in cooking
56203231 ^a^	Oatmeal, cooked, regular, made with milk, NS as to fat added in cooking
56203232 ^a^	Oatmeal, cooked, quick (1 or 3 min), made with milk, NS as to fat added in cooking
56203233 ^a^	Oatmeal, cooked, instant, made with milk, NS as to fat added in cooking
56203540	Oatmeal, made with milk and sugar, Puerto Rican style
56203600	Oatmeal, multigrain, cooked, NS as to fat added in cooking
56203610	Oatmeal, multigrain, cooked, fat not added in cooking
56203620	Oatmeal, multigrain, cooked, fat added in cooking
57804000 ^b^	Oatmeal cereal, baby food, dry, instant
57806100 ^b^	Oatmeal cereal with bananas, baby food, dry, instant
57806200 ^b^	Oatmeal cereal with fruit, baby food, dry, instant, toddler
57823000	Oatmeal with applesauce and bananas, baby food, jarred
67304500	Prunes with oatmeal, baby food, strained

Abbreviations: NHANES = National Health and Nutrition Examination Survey; NS = not specified; ^a^ Food codes were newly added to the 2009 to 2012 NHANES; ^b^ Dry oatmeal is cooked by adding 110 mL of water to every 15 g of dry oatmeal. Thus, the conversion factor applied to the dry oatmeal was 8.3.

**Table 2:** Total intakes of cooked oatmeal from all eating occasions among consumers of cooked oatmeal (data from Day 1 of the 2009–2012 NHANES).

Population Group (Years of Age)	Number of Individuals Surveyed	Consumers of Cooked Oatmeal					
		% Consumers	Number of Consumers	Intake of Cooked Oatmeal (g/Day)			
				Mean	90th Percentile	Min	Max
All Youths (0–18)	7407	4.6	412	182	330	3	844
Infants (0–2)	1863	14.3	264	148	285	3	844
Children (3–11)	3430	3.9	122	207	413	20	673
Female Teens (12–18)	1023	1.1	14	188	330	20	673
Male Teens (12–18)	1091	1.3	12	326	376	160	520
All Adults (19+)	10,866	6.5	708	251	424	5	1404
Female Adults (19–44)	2480	4.3	97	262	424	5	1404
Male Adults (19–44)	2382	3.1	67	316	489	132	936
Female Adults (45+)	3037	11.1	328	219	307	10	792
Male Adults (45+)	2967	6.7	216	277	424	29	1024
Total Population (all)	18,273	6.0	1120	238	424	3	1404

**Table 3:** Prevalence of consumption and intakes of cooked oatmeal at each eating occasion (data from Day 1 of the 2009–2012 NHANES).

Population Group (Years of Age)	Consumers of Cooked Oatmeal							
	Breakfast		Lunch		Dinner		Snack	
	Number of Consumers (%) ^b^	Mean Intake (g)	Number of Consumers (%) ^b^	Mean Intake (g)	Number of Consumers (%) ^b^	Mean Intake (g)	Number of Consumers (%) ^b^	Mean Intake (g)
**All Youths (0 to 18)**	**298 (3.7)**	**185**	**21 (0.2)**	**144**	**30 (0.3)**	**109**	**35 (0.3)**	**150**
Infants (0 to 2) ^a^	168 (9.6)	148	16 (0.8)	99	23 (1.3)	105	26 (1.2)	98
Children (3 to 11)	109 (3.7)	204	3 (0.1)	230	6 (0.1)	122	7 (0.1)	184
Female Teens (12 to 18)	12 (1.0)	190	1 (0.1)	176	1 (0)	176	0 (0)	0
Male Teens (12 to 18)	9 (1.1)	312	1 (0)	495	0 (0)	0	2 (0.2)	379
**All Adults (19+)**	**632 (5.8)**	**243**	**31 (0.3)**	**288**	**27 (0.1)**	**329**	**32 (0.3)**	**286**
Female Adults (19 to 44)	84 (3.9)	247	4 (0.2)	261	4 (0.1)	331	9 (0.3)	316
Male Adults (19 to 44)	55 (2.3)	302	3 (0.2)	300	5 (0.2)	390	5 (0.5)	362
Female Adults (45+)	296 (10.3)	215	14 (0.4)	261	12 (0.2)	270	11 (0.2)	196
Male Adults (45+)	197 (6.0)	273	10 (0.4)	330	6 (0.1)	413	7 (0.4)	224
**Total Population (all)**	**930 (5.3)**	**232**	**52 (0.3)**	**261**	**57 (0.2)**	**241**	**67 (0.3)**	**256**

^a^ It should be noted that there were 64 infants (3.0% of infants surveyed) for whom the eating occasion was defined as “infant feeding”; ^b^ Values in parentheses indicate the percentage of individuals surveyed who consumed oatmeal at the specific eating occasion on Day 1 of the NHANES (2009–2012). The total number of individuals surveyed in each age and gender group can be found in Table 2.

**Table 4:** The contribution of cooked oatmeal to the total intake of energy at breakfast in consumers of cooked oatmeal (data from Day 1 of the 2009–2012 NHANES).

Population Group (Years of Age)	Consumers of Cooked Oatmeal							
	Energy Intake at Breakfast (kcal)				Contribution of Cooked Oatmeal to the Intake of Energy at Breakfast (%)			
	Mean	90th Percentile	Min	Max	Mean	90th Percentile	Min	Max
**All Youths (0 to 18)**	**162.9**	**315**	**1.0**	**719**	**54.4**	**100**	**0.4**	**100**
Infants (0 to 2)	117.2	222	1.0	450	53.7	100	0.4	100
Children (3 to 11)	189.0	342	17.0	719	53.9	100	4.9	100
Female Teens (12 to 18)	192.2	300	63.0	576	44.2	77.1	13.6	77.1
Male Teens (12 to 18)	266.1	300	153.0	476	74.0	90.6	23.9	100
**All Adults (19+)**	**200.3**	**322**	**3.0**	**843**	**54.3**	**96.4**	**1.8**	**100**
Female Adults (19 to 44)	223.4	389	3.0	662	64.0	100	11.6	100
Male Adults (19 to 44)	267.1	453	96.0	623	59.2	98.8	11.6	100
Female Adults (45+)	174.5	287	18.0	657	52.3	90.5	1.8	100
Male Adults (45+)	211.8	328	31.0	843	50.6	95.3	4.3	100
**Total Population (all)**	**193.7**	**322**	**1**	**843**	**54.3**	**96.4**	**0.4**	**100**

**Table 5:** Foods co-consumed with cooked oatmeal at breakfast (data from Day 1 of the 2009–2012 NHANES).

Population Group (Years of Age)	Total Number of Oatmeal Breakfast Eating Occasions ^a^	Top Five Foods Most Frequently Consumed with Oatmeal at Breakfast ^b,c^	
		Food	Frequency of Consumption
All Youths (0 to 18)	310	Milk and yoghurt	210
		Fruit juices	90
		Fruits	86
		Water, noncarbonated	74
		Sugar and sweets	73
All Adults (19+)	669	Sugars and sweets	412
		Coffee and tea	355
		Fruits	323
		Milk and yoghurt	323
		Yeast breads, rolls, quick breads	165
Total Population (all)	979	Milk and yoghurt	533
		Sugars and sweets	485
		Fruits	409
		Coffee and tea	361
		Fruit juices	235

^a^ The total number of oatmeal breakfast eating occasions may be slightly larger than the total number of breakfast oatmeal consumers listed in Table 3 if a respondent reported more than one oatmeal eating occasion as breakfast on Day 1 of the 2009–2012 NHANES; ^b^ It should be noted that the number of breakfast eating occasions during which only cooked oatmeal was consumed was 24 for all youths (0–18 years), 13 for infants (0–2 years), nine for children (3–11 years), zero for female teens (12–18 years), two for male teens (12–18 years), 35 for all adults (19 years+), 12 for female adults (19–44 years), five for male adults (19–44 years), 11 for female adults (45 years+), seven for male adults (45 years+), and 59 for the total population; ^c^ The number of occurrences of other foods co-consumed with cooked oatmeal at breakfast was tallied.

**Table 6:** Foods most commonly consumed at breakfast in non-consumers of oatmeal (data from Day 1 of the 2009–2012 NHANES).

Population Group (Years of Age)	Total Number of Breakfast Eating Occasions	Top Five Foods Most Frequently Consumed in Non-Consumers of Oatmeal	
		Food	Frequency of Consumption
All Youths (0 to 18)	5700	Milk and yoghurt	4107
		Cereals (other than oatmeal)	2483
		Fruit juices	1651
		Yeast breads, rolls, quick breads	1388
		Sugar and sweets	1032
All Adults (19+)	8257	Coffee and tea	4636
		Milk and yoghurt	3796
		Sugars and sweets	3625
		Yeast breads, rolls, quick breads	3438
		Cereals (other than oatmeal)	2243
Total Population (all)	14,229	Milk and yoghurt	7903
		Coffee and tea	4863
		Yeast breads, rolls, quick breads	4826
		Cereals (other than oatmeal)	4726
		Sugars and sweets	4657

**Table 7:** Multiple linear regression analysis of the consumption of cooked oatmeal.

Independent Variables ^a^		Β (SE) ^b^	*p*-Value
NHANES Cycle		0.01 (0.03)	0.83
Age	3–11 years	5.35 (0.38)	<0.0001
	12–18 years	1.19 (0.37)	0.002
	19–44 years	0.36 (0.16)	0.03
	45 years+	---	---
Gender	Male	0.28 (0.14)	0.06
	Female	---	---
BMI	Underweight	3.01 (0.55)	<0.0001
	Normal weight	1.30 (0.13)	<0.0001
	Overweight	0.51 (0.12)	0.0001
	Obese	---	---

^a^ For age, age 45 years+ was used as the reference; for sex, female was used as the reference; for BMI, obese was used as the reference. The R-squared value for the model including all four independent variables is 0.39; ^b^ A positive value means a greater intake relative to the reference.
